# Benefits and challenges of electronic data capture (EDC) systems versus paper case report forms

**DOI:** 10.1186/1745-6215-16-S2-P37

**Published:** 2015-11-16

**Authors:** Ihtisham Malik, Stephanie Burnett, Mark Webster-Smith, James Morden, Sharon Ereira, Alexa Gillman, Rebecca Lewis, Emma Hall, Judith Bliss, Claire Snowdon

**Affiliations:** 1 The Institute of Cancer Research Clinical Trials & Statistics Unit (ICR-CTSU), London, UK

## Background

ICR-CTSU introduced EDC in 2012, requiring revision of systems developed for paper case report form (CRF) management. The challenges encountered and benefits realised with EDC are described.

## Challenges

EDC does not allow central data cleaning at point of entry or traditional manual CRF tracking upon receipt so alternative approaches are required. EDC specific site training is required and site staff need to be encouraged to submit data immediately following participant visits, instead of batching data entry. Database user access and internet browser compatibility monitoring systems are required to ensure only current site staff have access and are using a supported browser. Conventions for CRF design and submission require revision to facilitate EDC use at sites.

## Benefits

EDC allows logic checks to highlight discrepancies to site staff during data entry. Data can be reported in real time, ensuring safety data are readily available for central review, advantageous for high risk trials. Queries can be sent instantly with reduced turnaround times for data clarification. EDC provides an audit trail with data entry only by authorised individuals; challenging with CRFs if signatures are illegible. EDC also results in fewer transcription errors and illegible data issues, a frequent source of data queries on CRFs. Using EDC reduces paper management time and requires less physical storage space.

## Conclusion

The benefits of EDC outweigh the challenges however it requires continual reassessment and re-evaluation of novel processes as they are developed and implemented.

